# Increasing coping and strengthening resilience in nurses providing mental health care: Empirical qualitative research

**DOI:** 10.4102/hsag.v23i0.1094

**Published:** 2018-07-12

**Authors:** Rudor J. Ramalisa, Emmerentia du Plessis, Magdalena P. Koen

**Affiliations:** 1 Department of Health Science, Vaal University of Technology, South Africa; 2 Department of Health Science, North-West University, South Africa

## Abstract

**Background:**

Research on coping and resilience is on the rise. However, there is a paucity of information addressing strengths, assets, competence or resilience that enable nurses to remain committed and cope in their profession despite the adversities they face in their working environment.

**Objective:**

The purpose of this research was to explore and describe how to strengthen the resilience of nurses in a work environment with involuntary mental health care users.

**Method:**

An exploratory and descriptive research design, which is contextual in nature, was used.

**Results:**

Narrative responses to two open-ended questions (How do you cope with providing mental health care to involuntary admitted mental health care users? and; How can your resilience be strengthened to provide mental health care to involuntary mental health care users?) yielded coping mechanisms and resilience strengthening strategies.

**Conclusion:**

Nurses caring for involuntary mental health care users are faced with challenging situations while they themselves experience internal conflict and have limited choices available to be assertive. To strengthen their resilience, the following factors should be taken into account: support, trained staff, security measures and safety, teamwork and in-service training and education.

## Introduction

Nurses deal with adversities in their working environment that can leave them feeling demotivated, angry and dissatisfied, whereby some nurses choose to leave South Africa in search of greener pastures (Buchan 2006). Nurses play a pivotal role in the South African health care system, because a large proportion of the system is nurse based (Harrison 2009). Facing adversities, such as a lack of appreciation, poor remuneration, an insecure environment, a deficit in acknowledgement, exploitation, a sense of neglect and a lack of support, can compromise the quality of health care provided by nurses (Buchan 2006; Koen & Du Plessis 2011). In a context where mental health care users are admitted and treated involuntarily, nurses are exposed to stressors as well. Moreira and Loyola (2011) identified absconding and uncooperativeness, aggression, lengthy hospitalisation, emotional instability and suicide risks as facts that are evaluated by the mental health practitioners relating mental health users. The implication of this on nurses can be feelings of uneasiness, whereby nurses query whether they are acting in the best interest of these mental health care users.

As in many nursing specialist areas, nurses who provide mental health care also experience job dissatisfaction and adversities. It is therefore essential that nurses develop protective strategies to facilitate emotional toughness and modify the negative effects of adversities in order to strengthen their resilience (Hart, Brannan & De Chesnay 2014).

### Background

Nurses need the opportunity to develop resilient attributes in their different specialist areas. In her study on nursing students, Stephens (2013) proposed that developing, nurturing or enhancing resilience can be achieved through purposeful interventions and educational efforts to enhance protective factors (p. 130). Where nurses are not supported in developing these attributes, they can be compromised in their work and this can result in negative impacts on current retention rates as well as future recruitment (Gillespie et al. 2007). Individual resilience skills ensure that nurses are successful in their nursing career and that they can sustain themselves in challenging and difficult working climates (McGowan & Murray 2016).

Resilient nurses display distinctive qualities, such as intelligence, self-confidence, resourcefulness and flexibility (Brennan 2017). Resilience is a contextual and dynamic process (Aburn, Gott & Hoare 2015) that is ever-changing with the environment and is viewed as either a trait, a process or an outcome (Southwick et al. 2014). Masten (2014) defined *resilience* as the capacity of a dynamic system to adapt successfully to disturbances that threaten system function, viability or development. Aburn et al. (2015) identified five key themes of resilience, namely *rising above to overcome adversity*; *adaptation and adjustment*; *ordinary magic*; *good mental health as a proxy for resilience*; and *the ability to bounce back*.

Multiple concept analyses have been undertaken to explore resilience and its relevance to health care professionals (Aburn et al. 2015; Brennan 2017; Gillespie et al. 2007). One such study has shown that mental health care providers working in complex, stressful environments demonstrate resilience (Edward 2005). Aspects of resilience include a sense of self, faith, hope, an insight and looking after oneself (Edward 2005; Gillespie et al. 2007). Various attributes are associated with resilient behaviour – in order for individuals to be resilient, the risk factors related to mental health care should be understood.

Involuntary admissions of mental health care users at public health care facilities are on the rise (Lund et al. 2008). This is where the majority of patients are treated, yet the least resources are available (Coovadia et al. 2009). When involuntary admissions increase, the strain placed on nurses also increases. By and large, other factors contribute to this strain; the guidelines stipulate that nurses must obtain consent for care and treatment (Harrison 2009).

The rise in mental diseases in South Africa is reported in the complex environment of mental health as resulting from risk factors such as poverty, inequality, urbanisation, unemployment, trauma, violence and substance abuse (Burns 2011). About 50% of the South African population lives under the poverty line, whereas the unemployment rate is at 24% (Burns 2011). Other significant challenges in South Africa are the high crime and drug offence rates. The prevalence of HIV/AIDS in the country is estimated at approximately 18% and significantly increases the burden of neuropsychiatric diseases and disabilities, which can include depression, anxiety, psychosis and dementia (Burns 2011).

### Problem statement

Earlier research confirmed that psychiatric nurses identified caring for and treating involuntary mental health care users as emotionally upsetting (Basson 2012; Mavundla 2000). These negative feelings together with adopted attitudes have been viewed by patients as authoritarian, paternalist, intimidating and condescending (Borille, Paes & Brusamarello 2013; Moreno-Poyato et al. 2016). The argument according to Hem et al. (2018) is that coercive and paternalist treatment may improve the patient’s health; versus not doing what is seen as ‘good’ or ‘safe’, which can cause harm. Furthermore, this treatment usually results in tension in the balance between paternalism and autonomy in providing care. Moreira and Loyola (2011) highlighted that involuntary mental health care users can have a negative impact on nurse–patient relationships.

In order to care for mental health care users, Borille et al. (2013) stated that nurses must consciously ‘use the own person’ therapeutically. However, some feel they are not adequately equipped with the necessary knowledge to care for mental health care users (Mavundla 2000), especially if they have little or no mental health care training. According to Ross and Goldner (2009) they display fear of mental health care users and attach stigma to them. A lack of knowledge in caring for mental health care users can result in nurses developing a negative perception of their patients, resulting in compromised care (Mavundla 2000).

Another factor is the issue of safety and security when caring for involuntary mental health care users. Aggression and violence towards nurses are acknowledged as a source of stress for nurses providing mental health care (Mavundla 2000; Moreira & Loyola 2011). In addition to the growing challenges of caring for mental health care users – especially involuntary mental health care users – nurses perceive that the dignity of mental health care users is violated; the tendency is for these patients not to be taken seriously and they are ignored, physically violated and exposed (Gustafsson, Wigerblad & Lindwall 2014).

From the above-mentioned discussion, it is clear that nurses experience adversities while providing quality care to mental health care users, which can demotivate them. Strategies to enhance the resilience and identify coping mechanisms of these nurses remain to be investigated, as there is a paucity of such information. With these considerations in mind, this research therefore aimed to address the following questions: How do nurses cope while providing mental health care; and how can their resilience be strengthened in a work environment where mental health care users are often admitted involuntarily?

### Research purpose

This research aimed to explore and describe how to strengthen the resilience of nurses providing mental health care in order for them to provide quality nursing care in a work environment where mental health care users are often admitted involuntarily. The objectives of this research were to explore and describe how nurses cope while providing mental health care to involuntary mental health care users and to explore and describe how their resilience can be strengthened.

### Contribution to the field

This research was overarched by the RISE project (Strengthening the resilience of health caregivers and risk groups). Its aim is to explore and describe a multifaceted approach to strengthen the resilience of health care providers and at-risk groups (Koen & Du Plessis 2011). Within the RISE study, this research provides an exploration and description of the resilience of nurses and how they cope while they care for and provide treatment to involuntary mental health care users. This research also provides direction on how their resilience can be strengthened.

## Research design and method

### Design and context

To address the research questions, the researcher employed a methodology that revolves on the way in which the participants make sense of their subjective reality and attach meaning to it (Holloway & Galvin 2017). Thus an exploratory and descriptive research design was used, which is contextual in nature (Polit & Beck 2012).

The research project was done in a public mental health care facility in the North-West Province, South Africa. The psychiatric ward can admit approximately 120 male and female adult and juvenile mental health care users, of which the majority are involuntary admissions.

### Population and sampling

The research population was professional nurses (*n* = 32) working in a psychiatric ward for a period longer than three months. Participants had to be proportionate with the total number of nurses working in the ward in order for the results to be representative (Holloway & Galvin 2017). Twenty-four participants were able to take part in this research and the overall response rate was above target at 85.7% (*n* = 24). This population was selected in order to obtain information that can be provided by knowledgeable and experienced participants.

A convenience sampling method was used, as the participants’ ideas or experiences would help achieve the aim of the research (Holloway & Galvin 2017). Participation was voluntary and participants were recruited at the ward by word of mouth. The hospital nurse manager and operational managers acted as the intermediaries. An information session was conducted with the participants, during which the research was explained as well as what was expected of them.

### Data collection

Data collection for the research took place in 2013, by means of written narratives of two open-ended questions. Narratives allow the researcher to gain access to new perspectives, thoughts and experiences in order to analyse them in the context of personal stories and sense-making (Holloway & Galvin 2017).

Psychological and sociological contexts of the stories are taken into consideration, as they affect the storyteller (Saldana 2016).

The participants provided demographic information and wrote a narrative about the following two structured open-ended questions:

Please share your story by writing about the following:

- How do you cope with providing mental health care to involuntary mental health care users?
- How can your resilience be strengthened to provide mental health care to involuntarily admitted and treated mental health care users?

### Data analysis

In order to analyse the data from the participants’ written narratives and to ensure credibility of the study, the researcher used an independent co-coder (Polit & Beck 2012). Deductive thematic analysis was performed on each narrative response, based on the two questions set out to the participants (Riessman 2005). The narrative responses to each question were analysed and divided into separate groups, to develop sub-themes. Sub-themes shared similar notions whereby these recurring notions highlighted how the participants coped in providing care to involuntary mental health care users as well as strengthening their resilience.

## Defining key concepts

### Resilience

As a psychological concept, resilience embodies personal qualities that enable individuals to thrive when faced with adversities (Connor & Davidson 2003). According to Southwick et al. (2014), *resilience* is a stable trajectory of healthy functioning after a highly adverse event; a conscious effort to move forward in an insightful and integrated positive manner as a result of lessons learned from an adverse experience; the capacity of a dynamic system to adapt successfully to disturbances that threaten the viability, function and development of that system; and a process to harness resources in order to sustain well-being. Masten (2014) stated that the concept of resilience is applicable to many interacting levels; therefore this definition of *resilience* is applicable to nurses who are facing the adversities of providing nursing care to involuntary mental health care users.

### Involuntary mental health care users

*Involuntary mental health care users* refers to individuals who receive care, treatment and rehabilitation services or those who make use of health services at a health establishment aimed at enhancing their mental health status under Section 33 of the *Mental Health Care Act 17 of 2002*. Individuals are admitted and treated even though they have not consented to treatment – they are, therefore, protected from harming themselves, others or the property of individuals (Mental Health Care Act 2002).

## Results

Participants were recruited to the study on the inclusion criteria that they be registered nurses with psychiatric experience of longer than three months. The hospital nurse manager and unit managers facilitated the recruitment as mediators. The researcher provided each individual participant with a verbal explanation of the study. Those who responded to the narratives signed a consent form and provided their demographic data.

Overall there were 24 participants in the study, of whom the majority were female nurses. Their years of experience working in a psychiatric health care setting varied from two to eight years, whereas their level of education included a diploma in general nursing, diploma in psychiatric training, bachelor’s degree and postgraduate qualification.

Data saturation was evident in the theoretical thematic analysis of the content through grouping of the narrative responses. An analysis of the narratives and the identification of a common pattern in the responses of the participants yielded the following two main themes: *coping mechanisms* and *resilience strengthening*, as summarised in Table 1. A discussion of these themes and their sub-themes are presented in the following paragraphs. Each theme is discussed, followed by extracts from the narratives and linked to relevant literature control.

**TABLE 1:** The coping mechanisms of the participants and ways to strengthen resilience.

Themes	Coping mechanisms	Resilience strengthening
Sub-themes	Knowledge, skills and experience Nurse–patient relationship Support system Spirituality (religion) and self-care Coping with involuntary MHCUs is difficult and challenging	Support Trained staff Security measure and safety Teamwork and in-service training Education

MHCU, mental health care user.

### Coping mechanisms

The participants acknowledged that coping with the provision of care to involuntary mental health care users was difficult and challenging. As mentioned, this main theme emerged from questions and the participants’ responses to the narratives inferred these coping mechanisms. Effective coping and adaption, in this challenging environment, foster resilience when faced with this adversity. Despite the use of coping mechanisms, participants stated that coping with the treatment of involuntary mental health care users was difficult and challenging. The following were the responses of some participants:

‘It is not easy, because the mental health care user can sure be difficult’. (Participant 18)

‘It is difficult because sometimes they refuse treatment …’ (Participant 23)

‘It is very [difficult] to stay calm and positive …’ (Participant 7)

‘… at times it’s difficult …’ (Participant 12)

Factors such as violent and aggressive patients, patients refusing to undergo treatment and inability to communicate contribute to this. The participants viewed these situations as challenging but they also believed that these obstacles were opportunities available to them to provide quality care.

#### Sub-theme 1: Knowledge, skills and experience

Knowledge, skills and experience played a role in the nurses’ ability to cope while providing mental health care to involuntary mental health care users. Skills and knowledge enhance coping and can therefore be linked to the resilience of the nurse:

‘Skills and experience that I have acquired make me cope easily …’ (Participant 2)

‘Using skills to control them’. (Participant 23)

These skills were obtained in training or through ongoing in-service as these participants indicated:

‘Due to the training and skill obtained in my training and practical, I believe for me to cope …’ (Participant 11)

‘Also with help from your supervisor with in-service training …’ (Participant 20)

Cameron and Brownie (2010) found that resilience, amongst a group of registered nurses, was associated with clinical knowledge, skills and experience that could lead to to the competent, skilful and holistic care of patients. Itzhaki et al. (2015) indicated that even though some nurses may have received training in dealing with violence in mental health institutions, they can still feel inadequate to cope with violence. Training and developing skills in these nurses should therefore be frequent to promote coping mechanisms.

#### Sub-theme 2: Nurse–patient relationship

Some of the participants focused on their relationships and interactions with involuntary mental health care users as a coping mechanism. They loved providing care to their patients and went beyond their duty in order to alleviate suffering:

‘Most of the times you find yourself lying/bribing these users in order to get your work done …’ (Participant 24)

Communication, health education and explanation that facilitated nurse–patient relationships were viewed as coping strategies:

‘Throughout the admission period, health education should be emphasised …’ (Participant 11)

‘… to be able to explain to the patient the reason for them to drink medication …’ (Participant 1)

Trust and respect forms the foundation of the nurse–patient dyad (Moreno-Poyato et al. 2016). Patients sought to be treated as equals and be empowered in caring for themselves (Borille et al. 2013). Therefore, effectively communicating with patients enhances the relationship as correct information is transferred (Stenhouse 2010).

#### Sub-theme 3: Support system

Support includes a personal support system, fellow colleagues, organisational support and family. Workplace support provided by colleagues and the organisation is of key importance in the direct delivery of care as a lack of teamwork can compromise patient care. The following was provided by participants:

‘Involuntary patients are dangerous but because of the teamwork I can cope’. (Participant 7)

‘… lack of support from management in MHCU such as managing aggressive in-service training’. (Participant 8)

However, relying on support from outside the workplace can also play a crucial role in the ability of nurses to cope:

‘Have your own support system, to give daily encouragement’. (Participant 4)

‘… and support from your colleagues and family’. (Participant 20)

Gustafsson et al. (2014) emphasised professional relationships and a conducive work environment as important to empower nurses to care for involuntary mental health care users. When nurses are supported with developmental programmes they are able to build and strengthen relationships with their peers and to develop a support network within their organisation (Hart et al. 2014). Lastly, in the organisation, the nurse manager has an important leadership role of supporting the nurses to increase their motivation and improve care for the patients.

#### Sub-theme 4: Spirituality (religion) and self-care

Several of the participants emphasised spirituality and self-care as methods of coping. Although the empirical literature on spiritual nursing care provides theoretical, conceptual and operational definitions, its application in practice remains a challenge (Monareng 2012). Yet this sense of higher protection and rejuvenation enabled the participants to cope in this environment. The participants expressed these sentiments as to how they coped:

Religion:

‘God’s grace is new every morning … take regular breaks’. (Participant 4)

‘… and by praying to God that He should protect me …’ (Participant 16)

‘I also exercise so that I can be healthy mentally and see [things] in a positive way’. (Participant 16)

Koen et al. (2011) found that spirituality and a healthy lifestyle can be coping strategies used by nurses. Furthermore, Monareng (2012) stated that when doubts, anxieties and questions arise, nurses use their personal and spiritual resources to cope and face these. Many authors concurred with this notion that nurses who practise a religion, spirituality and faith have higher coping abilities (Cameron & Brownie 2010; Zander, Hutton & King 2010; Zheng et al. 2017). Lastly Gillespie et al. (2007) and Edward (2005) indicated that work–life balance and self-care build personal resilience to cope with workplace adversity.

### Resilience strengthening

In identifying the aspects that illuminate how the participants strengthen their resilience, an analysis of the narratives resulted in five sub-themes: support; sufficiently trained staff; security measures and safety; teamwork and in-service training; and education (see Table 1). Participants in this study mainly suggested professional (external) traits rather than personal (internal) traits, which strengthens resilience.

#### Sub-theme 1: Support

The participants indicated that their resilience could be strengthened by receiving support from management (organisation) and supervisors, support groups and peer support. Providing support to each other could alleviate workplace adversities. The following are examples from the narratives:

‘Prompt psychological support to personnel’. (Participant 1)

‘… support those who are injured emotionally, to have management that is understanding and supportive’. (Participant 16)

‘It does help a lot when one has a support group or someone …’ (Participant 24)

Zander et al. (2010) stated that resilience in nurses was promoted by non–work-related support and through support at work, which includes the multidisciplinary treatment team, the organisation, families, friends and colleagues. O’Connell et al. (2011) outlined these key elements of peer support in the workplace to provide the following: an independent forum to debrief and address work issues, reduces stress while it improves well-being, improves communication and enhances problem-solving skills. If peer cohesion is facilitated and promoted and educative support is offered, nurses manifest higher levels of hope in their work environment (Gillespie et al. 2007).

#### Sub-theme 2: Trained staff

Resilience strengthening can be attributed to adequately trained staff. Labonté et al. (2015) suggested that the total number of nurse emigranting elsewhere, equites to 20% of the total number working in the public sector.

The participants were of the view that their workload was overwhelming them and with sufficiently trained personnel, the workload could be minimised. Staff members should specifically be trained in psychiatric and mental health care:

‘Enough trained staff on mental health’. (Participant 15)

‘Increase in personnel working in the unit to minimised workloads …’ (Participant 1)

Professional development was identified by Edward (2005) as a way to strengthen and foster resilience of nurses. When the organisation (hospital) is faced with challenges and scarce resources, they can meet the demands by empowering nurses who aspire to practise autonomously and grow professionally. Quality care demands that nurses engage in lifelong learning to improve their knowledge and skills (Lautizi, Laschinger & Ravazzolo 2009).

#### Sub-theme 3: Security measures and safety

Improved safety and security in the workplace were identified by the participants as important in reassuring them. Proper security measures in a well-developed infrastructure can provide staff members with the reassurance that they are safe and ultimately enable them to thrive in their workplace. The following was mentioned by the participants:

‘Increase in security measures’. (Participant 1)

‘Security and safety measures be attended to’. (Participant 15)

Safety and security in a workplace contribute to the development of resilience in nurses and was identified in a number of research studies (Itzhaki et al. 2015; Koen et al. 2011). According to Wyder et al. (2017), nurses are believed to be able to ensure safety in an unpredictable and emotionally heightened environment. Furthermore, they stated that this deviates from and contradicts the role of nurses.

#### Sub-theme 4: Teamwork

Teamwork amongst multi-professional members, colleagues and supervisors enhanced the resilience of the participants. They stated that teamwork gave them the courage to share incidents with managers and lifted their morale:

‘Even the team spirit can help us have morale in providing a total care …’ (Participant 12)

‘Teamwork is important to me …’ (Participant 18)

Strategies to build resilience in nurses can include providing professional development programmes in teamwork and teambuilding (Hart et al. 2014). Workplace satisfaction is achieved with the nature and degree of interpersonal relationships with colleagues, resulting in a positive influence on patient care (Zheng et al. 2017). Edward (2005) stated that teamwork is the protective veneer with regard to the work stress of nurses.

#### Sub-theme 5: In-service training and education

With continual in-service training and education, the resilience of nurses can be strengthened. In-service training sessions should keep nurses updated of the latest developments. Learning opportunities should also be created. The participants expressed the following:

‘Education or in-service on [the] *Mental Health Act* and latest development …’ (Participant 15)

‘… the necessary facilities to teach those who don’t understand how to care for such a patient …’ (Participant 16)

Cameron and Brownie (2010) linked resilience as an outcome of the experience, complex skills and knowledge required to manage time, crisis situations, prioritise tasks and staff. Koen et al. (2011) observed this as a professional asset that should be ongoing to equip nurses with relevant knowledge and skills. Organisations consider the needs and encourage participation in training to reduce stress and actively implement solutions (Brennan 2017).

## Ethical considerations

In addition, the researcher ensured that the rights of the participants were protected. These rights included the right to self-determination and informed consent, the right to privacy, anonymity and confidentiality and the benefit of the research concerning participants (Holloway & Galvin 2017).

Informed consent was obtained before data were collected. The researcher explained the research to the participants, including the risk–benefit ratio, voluntary participation and that the participants could withdraw from participation at any time.

The participants were selected based on being information-rich individuals with regard to the topic of the research. They were informed that they could withdraw from the research at any time without being penalised. They were made aware of the availability of a counsellor for emotional support if needed.

Participants were recruited via the nurse manager of the ward where data were collected. She verbally informed the nurses about the research and invited them to participate. The raw data are stored on a password-protected computer and will be locked away in a file drawer for a period of seven years.

## Trustworthiness

The framework of Lincoln and Guba was used to guide this research process. Trustworthiness was ensured by applying credibility, transferability, dependability and confirmability (Holloway & Galvin 2017).

*Credibility* was ensured by collecting data from different participants to ensure various perspectives. The findings of the researcher are compatible with the perceptions of the nurses under study. *Transferability* was ensured by the representativeness of the sample to the population and was applied by providing a rich and thorough description of information concerning the participants, context and settings. A detailed description of the research methodology was also provided to ensure the applicability of the findings to other contexts and ultimately to provide other researchers with sufficient information to evaluate similarity in contexts. *Dependability* was achieved by describing the precise methods followed during the gathering, analysis and interpretation of the data to ensure that replication can be achieved when the research is conducted by other researchers. To ensure *confirmability*, raw data were used and notes were made and kept for auditing purposes. A dense description of the research process was supplied, the involvement of a co-coder was explained and supervision by experienced qualitative researchers was made use of. Furthermore, the research study underwent an examination to determine its trustworthiness.

## Discussion

The results of this study are consistent with the growing body of nursing literature on this topic (Benade, Du Plessis & Koen 2017; Cameron & Brownie 2010; Gillespie et al. 2007; Zander et al. 2010). The prevalence of common mental disorders is significantly higher in South Africa than in any other World Mental Health African country (Burns 2011). With an increase in admissions of involuntary mental health care users noted between 2007 and 2008, caring became compromised. This research explored and described how nurses cope while providing mental health care and how the resilience of these nurses can be strengthened while they treat involuntary mental health care users.

Of the nurses who participated in this study, more than half wrote narratives, which made the response rate reasonable and allowed for data saturation. It is impressive how the coping mechanisms of these nurses were closely related to aspects they viewed as strengthening their resilience. Support, teamwork and training or education were especially emphasised by them as mechanisms to cope and strengthen their resilience. Previous studies indicated that these coping mechanisms and resilience strategies coincide with certain competencies needed for resiliency, as listed by Kumpfer (1999), namely ‘spiritual, cognitive, behavioural, physical, affective and holistic’ competencies.

In order for nurses to perceive themselves as competent in their work, Gillespie et al. (2007) stated that nurses are more likely to experience competency if they are equipped with specialised knowledge and skills. In addition, Cameron and Brownie (2010) associated resilience in nurses who care for the elderly with clinical knowledge, skills and experience that lead to competent and skilful holistic care. Hart et al. (2014) indicated that programmes that target resilience-building behaviour in nurses assist them in managing their daily workplace challenges. Appropriate relationships can be achieved by enhancing communication between mental health care users and nurses. Nurses should practise good communication during their interactions with mental health care users to be able to understand their needs. When nurses do not plan and deliver appropriate care to patients and invest in building relationships with them, this can result in a negative impact on the ability of nurses to sustain resilient behaviour in their workplace (Cameron & Brownie 2010). It is important to emphasise that this relationship is built on trust and respect (Moreno-Poyato et al. 2016).

Another critical aspect the nurses identified in both themes was support. Organisational, peer, resource or family support enhances the resilience of nurses. Research also indicates that resilience can be attributed to a supportive environment (Cameron & Brownie 2010; Zander et al. 2010). Gillespie et al. (2007) concur with this statement – a supportive workplace can reduce the effects of stressors and can enhance hope as a variable of resilience. Physical or psychological support can include opportunities for nurses to self-reflect, debrief or validate themselves and to provide relief through humour. Moreover, team camaraderie fosters resilience (Brennan 2017; Cameron & Brownie 2010). The nurses used coping mechanisms in their workplace in the form of self-care and faith in a higher power. Furthermore, the authors stated that these healthy activities result in physical, emotional and spiritual developments and ultimately, restore balance in the lives of nurses.

The nurses also expressed various hardships associated with caring for involuntary mental health care users. Nurses sometimes struggle to build relationships and communicate with mental health care users to understand and meet their needs for various reasons (Moreno-Poyato et al. 2016). Mental health care users feel they are viewed as problems to be solved (Moreno-Poyato et al. 2016), and according to Moreira and Loyola (2011) they are viewed as being ‘severe’ with the emphasis on their hostility, agitation and aggression. Moreover, nurses in mental health care institutions tend to harbour negative attitudes and perceptions and often display negative behaviour directed towards patients with mental illness, which can have a negative influence on patients (Basson 2012). Negative influences can alter nurse–patient relationships and ultimately the resilience and coping mechanisms of nurses working in a challenging environment.

In an environment where nurses’ exposure to mentally ill patients is limited, Mavundla (2000) stated that they expressed feeling fearfulness, frustration and despair. If it is expected of nurses to care for involuntary mental health care users with insufficient staff and staff members who are not adequately trained, nurses can easily become demoralised. If the workload is not distributed equally and is coupled with an increased admission rate, nurses can feel overloaded. By increasing the workforce, the resiliency of nurses can be strengthened. In-service training and continual education are of the utmost importance to strengthen resilience. In-service training should focus on equipping nurses to build resilience and competence and to cope with challenges in their workplace. If these skills are not developed, mental health care institutions compromise care provision, which can have a negative impact on current staff retention and future recruitment (Gillespie et al. 2007).

Nurses undertaking a united stance in the workplace have displayed a better understanding of each other’s differences and roles in the ward (O’Connell et al. 2011). Stress is reduced and improved communication is achieved, whereby nurses are able to provide care with clear problem-solving abilities. Teamwork relates not only to nurses but also to the management structure, doctors and other mental health care providers. Gillespie et al. (2007) referred to collaboration, Cameron and Brownie (2010) highlighted cohesive working teams, while McCann et al. (2013) put emphasis on teamwork. It is therefore clear that teamwork should ideally be emphasised, because it adds to their protective veneer (Edward 2005).

Lastly, the nurses emphasised the importance of feeling safe in their work environment. The nurses identified safety and security as issues of concern because of mental health care users who are often uncooperative, aggressive and resistant to treatment (Mavundla 2000). To maintain safety and control, nurses often use physical and chemical restraints. Resilience can be strengthened by the support of management in this regard by ensuring the safety of nurses in their work environment. In addition, nurses should be trained in the necessary skills to manage aggressive mental health care users effectively.

Yet despite the positive attributes related to fostering coping and strengthening resilience, it is worth considering that there may be some less desirable elements of the ‘resilient’ character (McGowan & Murray 2016).

## Practical implications

Strengthening the resilience of nurses in their work environment can be achieved by enhancing nurse–patient relationships. When nurses build relationships and communicate effectively with mental health care users, holistic care can be provided.

Training and continual skills development can increase the competency and confidence of nurses. In turn, their resilience is also strengthened when they take pride in caring for involuntary mental health care users.

Another aspect that should be highlighted is the importance of support systems in a work environment. When nurses value the contributions made by each other and when they share experiences and knowledge with each other, mental health care users receive quality care. Furthermore, a supportive work environment enables nurses to cope with challenges when caring for involuntary mental health care users.

Lastly, when spirituality and exercise are encouraged and supported, nurses are able to cope with challenges in their work environment and their resilience is strengthened. Nurses are allowed the opportunity to rejuvenate and gain strength to cope and be resilient.

## Limitations of the study

Taking into consideration that this was a research project for a masters’ dissertation, the scope was limited and the data were collected in one setting with a relatively small sample. As a result, the amount of information provided by the participants was limited. If data collection took place by means of focus groups or individual interviews, for example more information and clarification could have been obtained. However, the research process and findings are comprehensively described and transferability was obtained. The practical implications were discussed to illustrate the value of this research study.

## Recommendations

Supervisors and managers should create learning opportunities with the aim to empower nurses with relevant skills in providing care to mental health care users.

Training should be provided to nurses as it promotes self-care and resilience. Assertiveness will enable nurses to thrive in challenging situations when faced with having to make paternalistic decisions. When nurses are self-confident, they tend to be able to handle unpleasant feelings and situations, because of their dedication to their work. Nurses should be encouraged to pursue further education and to develop skills – they have a responsibility to keep up to date with the latest trends concerning mental health and this can be achieved by continual professional development.

Support systems in the work environment of nurses should be strengthened when care is provided to involuntary mental health care users. Support is essential to build the morale of staff members and to create unity amongst them – responsibilities should be equally distributed amongst nurses.

Nurses should each be viewed as an asset – acting in the best interest of mental health care users – their strengths should be developed on a continual basis. Nurses should therefore have the opportunity to make contributions and share inputs in developing nursing policies.

The field of mental health nursing should attract adequate numbers of students as a career choice. Proper facilitation and guidance should equip students to enter a workplace where involuntary mental health care users are cared for with confidence. Continual education, skills development and specialisation in the field of psychiatric and mental health care should also be encouraged.

## Conclusion

The aim and objectives of this research – to explore and describe how nurses cope while providing mental health care to involuntary mental health care users, and how their resilience can be strengthened – were achieved. Findings suggest that when nurses make use of coping mechanisms and resilience strengthening strategies, lower risk factors are reported and nurses are able to render quality care.

Recommendations to strengthen the resilience of nurses providing mental health care to involuntary mental health care users should be formulated.
